# A Levonorgestrel-Releasing Intrauterine Device in the Right Upper Quadrant: A Case Report

**DOI:** 10.7759/cureus.87997

**Published:** 2025-07-15

**Authors:** Joshua M Cohen, Stephanie L Fegale, Tammy Phan, Emmelyn Samones, Paul Savino

**Affiliations:** 1 Emergency Medicine, Loma Linda University Medical Center, Loma Linda, USA; 2 Obstetrics and Gynecology, Loma Linda University Medical Center, Loma Linda, USA

**Keywords:** abdominal pain, case report, emergency department, intrauterine device, uterine perforation

## Abstract

Abdominal pain is a common emergency department (ED) presentation. Studies suggest that female patients represent a majority of ED patients with abdominal pain. Abdominal pain requires a broad set of differential diagnoses, including gynecologic and obstetric causes. We describe the case of a patient who presented to the ED for right lower quadrant discomfort after the post-partum placement of a levonorgestrel-releasing intrauterine device (IUD). ED imaging revealed an IUD in the right upper quadrant. On laparoscopy, an intra-omental IUD was found near the hepatic flexure.

## Introduction

Abdominal pain historically accounts for at least 23 million or 5% of the total emergency department (ED) visits annually in the United States [1,2]. It has been suggested that women may represent a disproportionate number of the broader patient population with acute abdominal pain. Some estimates suggest that three in every four patients with abdominal pain in the ED are women [1]. For the emergency physician, women are a special patient population with their own unique pathology, risk factors, morbidity, and mortality. This special consideration is especially relevant for those of reproductive age. 

Classically, a strong emphasis has been placed on the pregnancy status of women undergoing evaluation for abdominal pain in the ED. While pregnancy status is important, research suggests that a more nuanced perspective is indicated, as significant maternal mortality in the postpartum period has been described both globally and in the United States [3]. Our case report hopes to increase awareness of an underdocumented and atypical cause of morbidity and mortality in a postpartum female with geographic and financial barriers to healthcare who underwent a routine procedure for the placement of a long-acting reversible contraceptive device.

## Case presentation

A 31-year-old gravidity 3 parity 3 (G3P3) female patient with class II obesity and prior ovarian cystectomy presented to an outside community health center in a medically underserved region for interval placement, four to eight weeks post-spontaneous vaginal delivery, of a levonorgestrel-releasing intrauterine device (IUD). Prior to this IUD placement at her initial postpartum visit, the patient had previously used an IUD without experiencing any common time of insertion to post-insertion complications (e.g., syncope/vagal reactions (2%), difficult insertion (20%), pregnancy (0.8%), expulsion/difficult removal/perforation). She did not experience significant pain, bleeding, or other complications at the time of placement. Insertion was completed without the use of ultrasonography, consistent with most IUD insertions in the absence of an anticipated difficult placement [4,5].

The patient presented to the clinic for a follow-up visit secondary to new-onset right lower quadrant pain four weeks later. She underwent pelvic ultrasonography at that time, which did not demonstrate the presence of an IUD. The patient was discharged with return precautions for suspected IUD expulsion. However, she presented one week later to another outside community clinic secondary to persistent symptomatic discomfort. Plain film radiography at that point demonstrated a suspected intra-abdominal foreign body, prompting an outpatient referral to a gynecologist for further evaluation. During the preoperative planning for a scheduled non-emergent diagnostic laparoscopy, bedside ultrasonography found a pericolic foreign body, for which the patient was referred to a tertiary medical center for emergent evaluation. 

**Figure 1:**
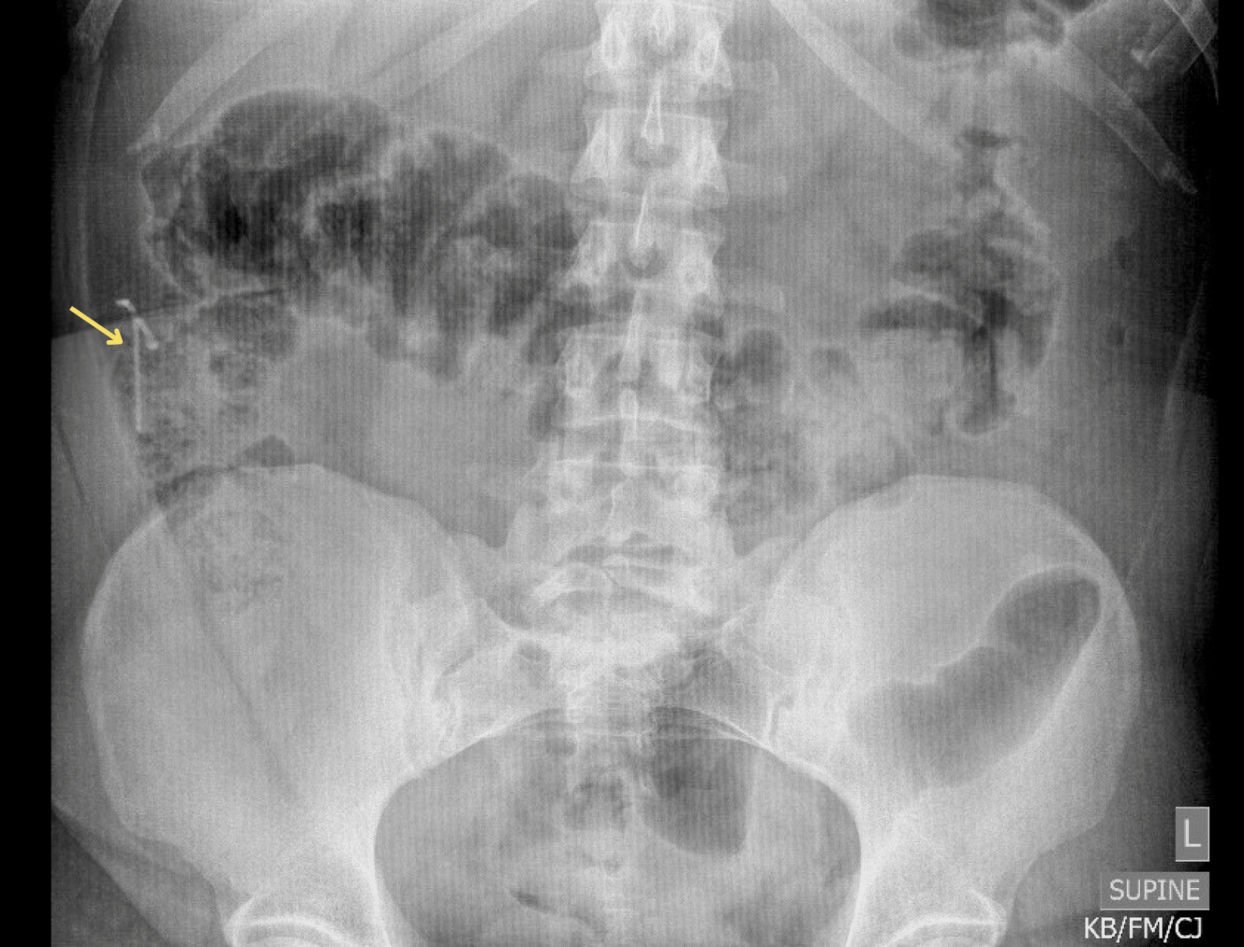
Plain radiograph of the abdomen and pelvis with the yellow arrow denoting an intra-abdominal foreign body

**Figure 2:**
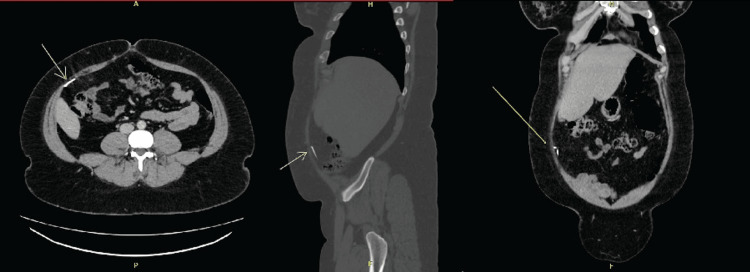
Computed tomography scan of the abdomen and pelvis with contrast with the intra-abdominal foreign body denoted by a yellow arrow in transverse, sagittal, and coronal sections

On presentation to the ED, the patient was hemodynamically stable and afebrile, with age-appropriate vital signs except moderate hypertension at 156/101 millimeters of mercury and a pulse rate of 95 beats per minute. She denied fever, chills, chest pain, dyspnea, nausea, emesis, increased abdominal girth, early satiety, vaginal discharge, or changes in gastrointestinal/genitourinary function. Normal monthly menses were reported. On exam, the patient was found to be uncomfortable appearing, though in no acute distress. She did, however, exhibit mild right lower quadrant tenderness to deep palpation with an otherwise reassuring pelvic examination. The patient had normal-appearing external genitalia with vaginal walls and cervix free of lesions, blood, abnormal discharge, or foul smell. Similarly, the cervix was smooth and intact without visible defects, though no IUD strings were found on sterile speculum exam. Bimanual examination found the cervix to be without nodularity, masses, or tenderness. Initial workup included a complete blood count, metabolic panel, coagulation studies, type and cross, lipase, and point-of-care pregnancy testing. Additional studies included plain film radiography and computed tomography (CT) imaging of the abdomen and pelvis with contrast (Figure 1 and Figure 2).

**Figure 3:**
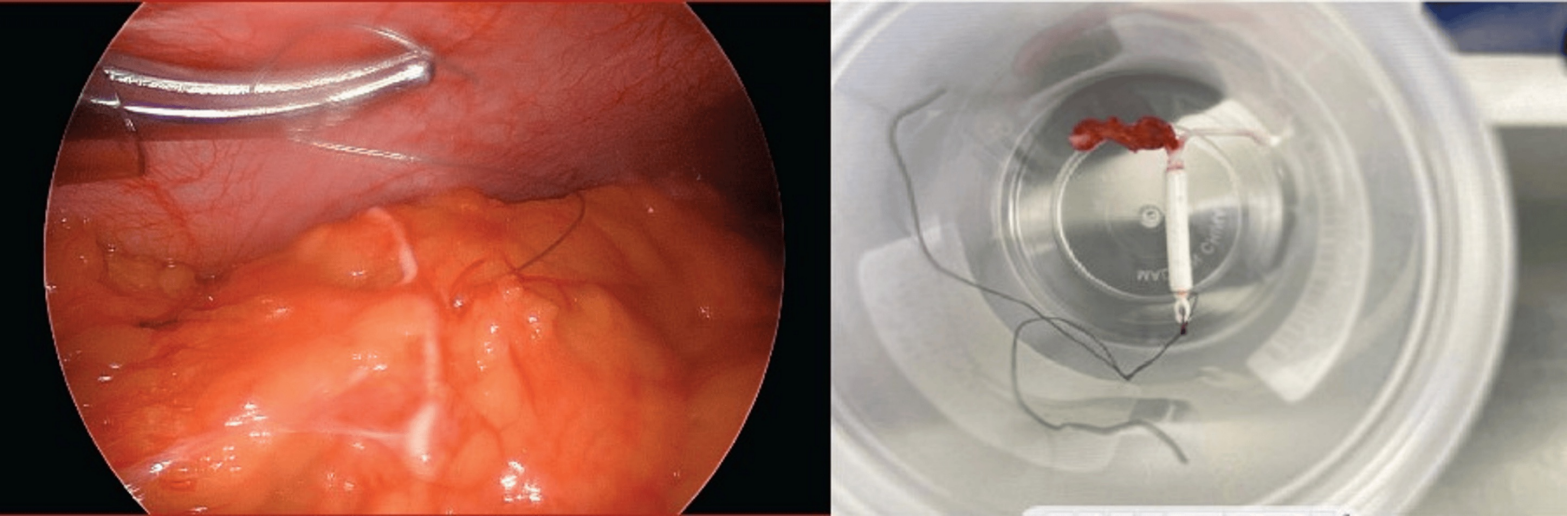
Intraoperative picture (left) from diagnostic laparoscopy of the intra-abdominal foreign body with the postoperative specimen (right) removed from the omentum consistent with likely intrauterine device

Initial laboratory studies returned without acute abnormality. Given concern for an intra-abdominal foreign body suspected to be secondary to a perforated IUD, gynecology was urgently consulted. The patient consented and was prepped for same-day urgent diagnostic laparoscopy. Laparoscopy identified a suspected intact IUD found encased in the omentum (Figure 3). Given its proximity to the bowel, acute care general surgeons provided intraoperative assistance in removal. As pictured in Figure 3, an intact IUD was removed from the omentum. Follow-up pathology confirmed the foreign body to be the initial IUD placed postpartum.

## Discussion

Between 2017 and 2019, approximately 10% of women in the United States used a long-acting reversible contraceptive, of which IUDs represent one type [6]. In the United States, five varieties of IUDs are currently approved by the Federal Drug Administration for contraceptive use in women, including those that are levonorgestrel-based (Kyleena, Liletta, Mirena, Skyla) and those that are copper-based (ParaGard). Each has varying attributes, from size, hormonal dose, and longevity, that may be particularly favorable for individual patients [7]. Regardless of the specific IUD preferred, the devices have similar safety profiles, indications, and contraindications.

Despite a wide availability and range of IUDs, many social determinants of health affect the reproductive wellness of patients seeking contraceptive care, particularly in the community health setting. One study in California alone suggested that community healthcare centers may lack the trained physicians, equipment, protocols/workflows, and financial incentives sufficient for expeditious and safe IUD placement despite a relatively overwhelming positive attitude towards its use by physicians and patients [8]. Whether any one of these factors may have impacted the suboptimal outcome and delayed definitive care in our patient remains unclear. Further research will be needed to clarify whether community health centers that place IUDs and other long-acting reversible contraceptives offer adequate resources or policies to troubleshoot the rare, though ever-present, potential adverse outcomes associated with these devices.

One particularly important, albeit uncommon, potential adverse outcome of IUD placement includes uterine perforation. It has been estimated that uterine perforation may occur once in every 1,000 IUD insertions, representing a 0.1% incidence [7,9]. This may be a primary outcome of poor insertion technique, difficult placement secondary to uterine anatomy or pathology, or the result of progressive uterine wall erosion [10]. Some studies have even begun to suggest that the timing of interval placement may account for the increased risk of perforation [11]. Regardless of the risk factors, uterine perforation secondary to remote IUD placement with subsequent migration remains a rare and oft-forgotten cause of abdominal pain in the ED patient.

Since the early 1980s, various techniques have been described for the initial evaluation of a missing IUD or suspected perforated uterus. These included plain film radiography, sonography, hysterography, hysteroscopy, CT imaging, or laparoscopy. No single technique has been definitively shown to be superior, and the choice may depend on clinician preference, clinician expertise, or consultation with a radiologist [12,13]. Though case reports of IUD insertion and perforation have been noted previously in the literature, many describe a copper-based or unspecified IUD type rather than a hormonal device. Similarly, many reports to date represent perforation in pregnant patients, a known risk factor [14-16]. Previously reported cases of a levonorgestrel-releasing IUD in the right upper quadrant or in the omentum involved patients with significant other gynecologic comorbidities (e.g., endometriosis) and long-term placement of an IUD rather than the rapid progression of migration observed in our patient, which occurred in less than eight weeks and was documented by serial radiography [17,18]. Given the possible rapid progression of immediate or delayed uterine perforation, as our case demonstrated, it remains imperative that emergency physicians maintain a high index of suspicion for atypical causes of abdominal pain.

## Conclusions

IUD perforation is an unfortunate and rare side effect of the use of this contraceptive device but an often missed or unrecognized cause of pelvic pain in the emergent setting. While a thorough gynecologic history can help identify this risk in the emergent setting, this case underscores the complexity and rarity of contraceptive complications, particularly in the vulnerable postpartum population. Our case highlights the need for enhanced training programs and experience for healthcare physicians who are placing these devices, as well as greater resources available to smaller clinics in the event of complications. Similarly, our case also exposes the need for developing refined guidelines and policies that ensure there is a systematic and reliable plan in place to help identify the potential for perforation, as this can happen to even the most experienced of physicians. With specific guidelines in place, clinics can improve the early detection of IUD malpositioning or perforation and decrease the risk of further complications or adverse outcomes experienced by patients. Improved protocols and awareness can also streamline patient care and decrease the number of healthcare centers patients must visit to obtain definitive care and diagnosis. Through a concerted effort to understand and mitigate the risks, healthcare physicians can ensure that the IUD remains a safe and effective form of postpartum contraception while reducing the risk of rare but serious complications.
